# One-step selection of high-affinity COP1 aptamers

**DOI:** 10.1016/j.abiote.2026.100060

**Published:** 2026-06-04

**Authors:** Lihong Song, Tiantian Zhou, Miqi Xu, Kaiqiang Qian, Xing Wang Deng, Qingqing Wu, Jun-Jie Ling

**Affiliations:** a School of Life Sciences, National Engineering Laboratory of Crop Stress Resistance Breeding, Anhui Agricultural University, Hefei, 230036, China; b State Key Laboratory of Wheat Improvement, Peking University Institute of Advanced Agricultural Sciences, Shandong Laboratory of Advanced Agricultural Sciences at Weifang, Weifang, 261000, China; c National Engineering Laboratory of Crop Stress Resistance Breeding, Anhui Agricultural University, Bio-breeding Laboratory of Anhui Province, Hefei, 230036, China

**Keywords:** COP1, Aptamer, One-step selection, ELONA, Plant synthetic biology

## Abstract

Aptamers are single-stranded DNA or RNA molecules that specifically bind to a wide range of target molecules with high affinity, making them powerful tools for synthetic biology. Traditional aptamer selection via Systematic Evolution of Ligands by Exponential Enrichment (SELEX) is labor-intensive and prone to nonspecific binding. To address these issues, we developed the Bead-based One-Step aptamer Selection (BOSS) method. As proof of concept, we used this method to target *Arabidopsis thaliana* CONSTITUTIVE PHOTOMORPHOGENIC 1 (AtCOP1), a conserved E3 ubiquitin ligase central to photomorphogenesis. We expressed the N-terminal RING (Really Interesting New Gene) domain of AtCOP1, immobilized it on Ni-NTA beads, and incubated the beads with a random ssDNA library. After washing the beads with washing buffer and replacing the buffer five times, followed by high-throughput sequencing, we identified the high-affinity aptamer Lib1-9, with a Kd of 11.14 nM for AtCOP1-RING. Lib1-9 demonstrated species specificity, showing strong binding to AtCOP1, but not to other COP1 homologs. Truncation analysis revealed that the core variable region (△LR) of Lib1-9 retained near-full binding affinity (Kd = 1.679 nM) to AtCOP1, which is comparable to the values observed for thrombin-binding aptamers. When we delivered Cy5-labeled aptamers into the hypocotyl cells of transgenic YFP-NLS-AtCOP1/*cop1-4 A. thaliana* plants, both Cy5-Lib1-9 and Cy5-Lib2-11 colocalized with YFP-COP1. The BOSS method is an efficient platform for plant aptamer development, enabling the rapid generation of tools for synthetic biology applications such as COP1 biosensing and optogenetic control.

*Dear editor*,

Aptamers are single-stranded DNA or RNA molecules with high affinity and specificity for a wide range of target molecules [1,2]. Aptamers are generally short-stranded and can thus be easily modified to serve as biosensors to detect biomarkers in clinical settings, engineered as genetic circuit tools for synthetic biology, or utilized as biosensors of small phytoactive molecules such as *trans*-zeatin and abscisic acid [3,4]. Despite their great potential, the traditional Systematic Evolution of Ligands by Exponential enrichment (SELEX) method for selecting aptamers is often laborious and time-consuming [5], as SELEX involves multiple rounds (10 to 15) of selection and amplification. To overcome these limitations, the one-step screening technique hydrogel for aptamer selection (HAS) was recently developed; HAS reduces the number of selection rounds and minimizes nonspecific binding [6]. However, few aptamers specific for plant proteins are currently available.

CONSTITUTIVE PHOTOMORPHOGENIC 1 (COP1) was first identified as a central repressor of photomorphogenesis in *Arabidopsis thaliana*. This well-studied protein (with over 1000 publication records from NCBI PubMed search) is an E3 ubiquitin ligase that is evolutionarily conserved from plants to animals [7,8]. To develop aptamers against plant proteins, we chose *A. thaliana* COP1 (AtCOP1) as proof of concept. Due to the key roles of AtCOP1 and the application of UV RESISTANCE LOCUS 8 (UVR8)-AtCOP1 in optogenetics [9,10], the selection of AtCOP1 aptamers would advance photobiology and provide useful tools for plant synthetic biology and optogenetics.

AtCOP1 contains three important domains required for its activity: an N-terminal Really Interesting New Gene (RING) domain (52–90 aa) for interactions with the ubiquitin-conjugating enzyme (E2), a middle coiled-coil domain (138–161 aa) for interactions with partners, and a C-terminal WD40 domain (342–675 aa) for interactions with substrates [11]. Since the RING domain is the key domain for the E3 ligase activity of AtCOP1, we constructed a plasmid expressing His-tagged AtCOP1-RING (N128) and purified His-AtCOP1-RING using Nickel-Nitrilotriacetic Acid (Ni-NTA) beads (Fig. S1). We then synthesized a Fam-labeled single-stranded DNA (ssDNA) library (∼N40) and incubated it with His-AtCOP1-RING prebound with Ni-NTA magnetic beads in binding buffer. Ni-NTA beads without proteins were also incubated with the random ssDNA library as a negative control. We collected the Ni-NTA beads, washed them with washing buffer, and replaced the washing buffer five times at the indicated time points, over a total of 60 hours (Fig. 1A and S2). After the last washing step, the DNA was eluted from the Ni-NTA beads and analyzed using fluorescence and gel electrophoresis (Fig. 1A, S2, and S3). We named this method, which uses rationally designed bead-based binding for one-step aptamer selection, Bead-based One-Step aptamer Selection (BOSS).

We collected the aptamer pools with two independent replicates (Lib1 and Lib2) from the Ni-NTA beads with or without His-AtCOP1-RING at 60 h, subjected them to next-generation sequencing, and analyzed the data on the Galaxy webserver (https://usegalaxy.org/). After filtering for sequence length, when Ni-NTA beads lacking His-AtCOP1-RING were utilized, no sequences were acquired from Lib1 or Lib2. By contrast, in the presence of His-AtCOP1-RING, 3447 sequences from Lib1 and 7487 sequences from Lib2 with were obtained after filtering out sequences that were inconsistent with the library in terms of length and fixed regions. We selected the top 50 sequences based on their abundance and sequence similarity (Fig. 1B, S4 and Table S1). Four candidates from Lib1 and three candidates from Lib2 were chosen and synthesized based on sequence abundance and predicted structural stability (Gibbs free energy) (Fig. 1B and S5). Two candidates showed a retention ratio comparable to that of the initial library, and the five remaining candidates exhibited higher binding activities (Fig. S5).

We chose one candidate from each library (Lib1-9 and Lib2-11) for an Enzyme-Linked OligoNucleotide Assay (ELONA) (Fig. 1C) [2] to measure their binding affinities to COP1-RING. Both aptamers exhibited high-affinity binding to recombinant COP1-RING, with Kd values of 11.14 and 3.741 nM for aptamers Lib1-9 and Lib2-11, respectively (Fig. 1D and S6). Therefore, Lib1-9 exhibited a stronger binding signal. We also examined whether Lib1-9 would show binding activity to full-length COP1. We purified full-length AtCOP1 (Fig. S7A) and determined that its affinity with Lib1-9 was stronger than that of AtCOP1-RING (Fig. 1D and S7B).

**Fig. 1:**
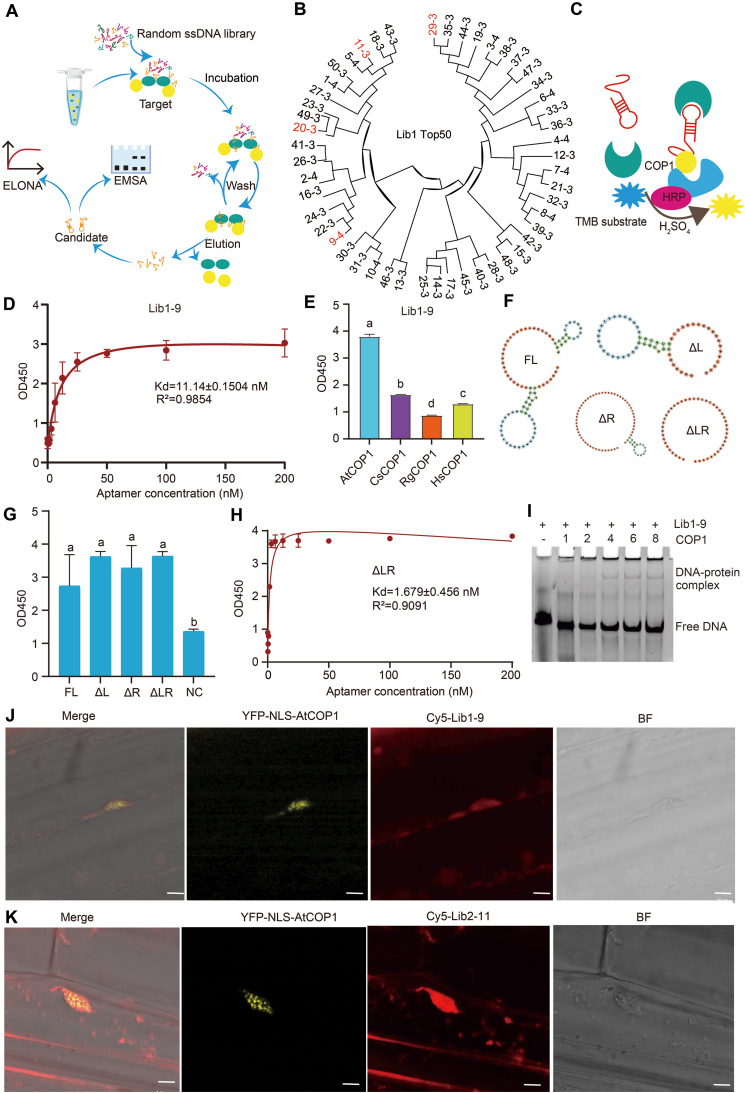
Selection and analysis of COP1 aptamers. **A** Selection scheme for AtCOP1 aptamers. AtCOP1 is anchored to Ni-NTA magnetic beads and incubated with a random ssDNA library with ∼40-mer oligonucleotides. The magnetic beads bound with COP1 and the library are washed in washing buffer for 60 h, with the washing buffer replaced every 12 h, for a total of five replacements, retaining aptamers with high-affinity binding to AtCOP1. The binding affinity and specificity of these aptamers are determined by enzyme-linked oligonucleotide assay (ELONA) and gel-shift assays. **B** Phylogenetic tree of the Top 50 aptamer candidates in Lib1. The candidates shown in red were chosen for further analysis. **C** Diagram of the ELONA. TMB: 3,3′,5,5′-tetramethylbenzidine. **D** ELONA examining the binding of Lib1-9 to AtCOP1-RING. The aptamer concentrations were 0, 0.20, 0.39, 0.78, 1.56, 3.13, 6.25, 12.5, 25, 50, 100, and 200 nM. **E** Species specificity of Lib1-9. ELONA examining the binding of Lib1-9 to COP1 homologs from *A*. *thaliana* (At), *R*. *globosum* (Rg), *C*. *sinensis* (Cs), and *H*. *sapiens* (Hs). Data are mean ± SD (*n* = 3). Statistical analysis was performed by one-way ANOVA with Brown-Forsythe and Welch's test (significance was set at ∗*P* < 0.05). Different lowercase letters above columns indicate statistical differences at *P* < 0.05. **F** The secondary structures of full-length (FL) and truncated versions of Lib1-9 predicted by the Mfold webserver. ΔL (16–89 nt), ΔR (16–89 nt), ΔLR (16–64 nt). **G** ELONA examining the binding of truncated versions of Lib1-9 to AtCOP1. Statistical analysis was performed by one-way ANOVA with Brown-Forsythe and Welch's test (significance was set at *P* < 0.05).Different lowercase letters above columns indicate statistical differences at *P* < 0.05. **H** ELONA examining the binding affinity of Lib1-9-ΔLR to AtCOP1-RING. The aptamer concentrations were 0.20, 0.39, 0.78, 1.56, 3.13, 6.25, 12.5, 25, 50, 100, and 200 nM. **I** Gel-shift assay of Lib1-9 with AtCOP1-RING. FAM-labeled Lib1-9 (1 pmol) formed a complex with AtCOP1-RING. Free DNA and DNA-protein complexes are indicated. **J**-**K** Epifluorescence microscopy of Cy5-Lib1-9 **(J)** and Cy5-Lib2-11 **(K)** with YFP-NLS-AtCOP1 in transgenic YFP-NLS-AtCOP1/*cop1-4Arabidopsis* hypocotyl cells. Seedlings were grown in the dark for 4 d and incubated with 10 μM Cy5-Lib1-9 or Cy5-Lib2-11 for 12 h. The scale bar is 10 μm. BF: bright field.

Furthermore, we purified three COP1-RING proteins from three other species, *Rhizoclosmatium globosum* (a fungus), *Camellia sinensis* (tea plant), and *Homo sapiens* (human) and analyzed the binding specificity of Lib1-9 to these proteins (Fig. S8). A comparison of COP1-RING proteins among *A*. *thaliana* (At), *R*. *globosum* (Rg), *C*. *sinensis* (Cs), and *H*. *sapiens* (Hs) showed that Lib1-9 displayed the highest binding specificity with AtCOP1-RING (Fig. 1E). Thus, we successfully identified an aptamer with high affinity and specificity for AtCOP1 through one-step selection.

The predicted full-length secondary structure of Lib1-9 contains multiple stem-loops (Fig. 1F). Based on this predicted secondary structure, we truncated Lib1-9 to generate three shorter aptamer sequences (ΔL, ΔR, ΔLR) with different secondary structures (Fig. 1F and Table S1) and examined their binding affinities using an ELONA. All three truncated aptamers exhibited high binding affinities for AtCOP1-RING (Fig. 1G), indicating that the core variable region (ΔLR) of Lib1-9 retained robust binding affinity for AtCOP1-RING. ELONA revealed that Lib1-9-ΔLR, with only an N40 variable region, had higher affinity for AtCOP1-RING, with a Kd of 1.679 nM (Fig. 1H), which is comparable to the binding affinity of DNA aptamer 60.29 for thrombin (Fig. S9), a classic target of aptamers [12].

To further confirm the interaction between Lib1-9 and AtCOP1, we performed a gel-shift assay using Lib1-9 fluorescently labeled with Fam. Adding increasing amounts of recombinant AtCOP1-RING protein to Fam-Lib1-9 led to an enhanced gel shift, indicating that Lib1-9 physically interacts AtCOP1-RING (Fig. 1I).

Since COP1 translocates from the nucleus to the cytoplasm after exposure to the light [13,14], we synthesized Cy5-labeled aptamers and delivered them into the hypocotyl cells of transgenic 4-day-old *A. thaliana* YFP-NLS-AtCOP1/*cop1-4* seedlings [15]. At 12 h after delivery, both Cy5-Lib1-9 and Cy5-Lib2-11 colocalized with YFP-NLS-AtCOP1 in the nuclei of hypocotyl cells (Fig. 1J and K), whereas there was no significant overlap between the aptamers and 35S-GFP signals in the *35S-GFP/Col-0* control (Fig. S10). The colocalization of COP1 and the aptamers was observed in most but not all cells, indicating that these aptamers could be further optimized for use as biosensors.

In summary, we developed specific aptamers for AtCOP1, providing promising tools for use with light-based synthetic biology devices. The BOSS method used in this study offers a rapid and efficient alternative to traditional SELEX methods, reducing the time and resources required for plant aptamer selection. In addition, the high affinity and specificity of the newly identified aptamers make them efficient tools for the subcellular localization of AtCOP1 (Fig. 1J and K). It would be worthwhile to further optimize these aptamers via directed evolution to enhance their sensitivity as *in vivo* biosensors. Finally, it would be interesting to explore whether AtCOP1 affects gene expression in plant cells directly through binding target-gene promoter regions that resemble the aptamer sequences. Overall, we conclude that the BOSS method provides a practical platform for identifying potentially valuable aptamers, with great promise for use in plant synthetic biology and optogenetics.

## 1 Materials and methods

### 1.1 Materials

Lib1 and Lib2, with random ∼ N40 sequences flanked by fixed primer-binding regions, were synthesized in two independent batches by Tsingke company using two different random libraries. The aptamer library and primer sequences are listed in Table S2. Human thrombin was purchased from Prolytix Haemtech. The DNA gel extraction kit and plasmid extraction kit were purchased from OMEGA. High-fidelity DNA polymerase 2× Master Mix was obtained from Vazyme. Ni-NTA magnetic beads were purchased from Thermo Fisher Scientific.

Seeds from *A. thaliana 35S-YFP-NLS-COP1/cop1-4* plants [15] were a gift from Ruohe Yin, and seeds from *35S-GFP/Col-0* plants were a gift from Dr. Chao Xu.

### 1.2 Construction and purification of COP1s

The optimized coding sequences of *RgCOP1*-RING and full-length *AtCOP1* were synthesized by Tsingke Company. The coding sequences of *AtCOP1*-RING, *HsCOP1*-RING, and *CsCOP1*-RING were amplified from *A. thaliana, H. sapiens,* and *C. sinensis* cDNA*,* respectively*.* These coding sequences were inserted into pET-28a using the assembly method. Exogenous protein expression in *E. coli* strain BL21 (DE3) carrying these constructs was induced by adding 0.5 mM isopropyl β-d-thiogalactopyranoside. The cells were harvested by centrifugation, resuspended in 1× PBS, lysed by sonication, and purified using Ni-NTA.

### 1.3 One-step aptamer selection

The process used for aptamer selection involved the following steps: Ni-NTA magnetic beads were pre-washed and incubated with or without 10 μg of AtCOP1-RING in binding buffer (1× PBS 7.4, 5 mM MgCl_2_, 1 mM CaCl_2_, 2.5 mM KCl) at 4 °C for 30 minutes. The beads were washed three times with washing buffer (1× PBS, 0.2% Triton-X, 300 mM NaCl) to remove non-specifically bound proteins. The ssDNA library (100 pmol) was denatured at 95 °C for 5 minutes, slowly cooled to 4 °C, and added to the protein-bound beads. The mixture was incubated at 4 °C for 60 h in washing buffer, with the washing buffer replaced every 12 h for a total of five times. After the last buffer replacement, bound aptamers were eluted at 95 °C for 5 minutes, and the eluted aptamers were amplified by PCR with specific primers. The amplified products were gel-purified and analyzed by next-generation sequencing.

### 1.4 Next-generation sequencing and bioinformatics analysis

Gel-purified DNAs were sequenced using the Illumina next-generation sequencing platform by Biozeron Biotechnology. The raw data obtained in fastq format were imported into the Galaxy webserver (https://usegalaxy.org/) in standard format. Low-quality sequences were discarded using the FASTQ Groomer tool. The clean sequences were sorted by Barcode Splitter to sort barcoded data with barcodes and consistent 5′ regions. The sequences were further filtered by length (80–100 nt). Reads with unique sequences were then identified using the “collapse” tool. Finally, the top 50 candidates based on sequence reads were identified as potential aptamers for AtCOP1-RING. Multiple sequence alignment of these sequences was performed using MEGA to identify sequence families. Based on the reads of sequence families and the Gibbs energy calculated by mFold (http://www.unafold.org/mfold), candidates were chosen for synthesis and further analysis.

### 1.5 Enzyme-Linked OligoNucleotide Assay (ELONA)

An enzyme-linked assay to determine the binding activities of biotinylated aptamers to COP1 was performed as described previously, with some modifications [2]. A 96-well high-binding polystyrene microtiter plate was coated with recombinant proteins (100 nM, final concentration) in 200 μL of binding buffer at 4 °C for 16 h. After washing three times with washing buffer (1× PBS, 0.2% Triton-X, 300 mM NaCl), the plate was blocked with blocking buffer (1× PBS, 0.2% Triton-X, 300 mM NaCl, 2% bovine serum albumin) and incubated for 30 minutes at 37 °C. Biotin-labeled aptamers (0.20, 0.39, 0.78, 1.56, 3.13, 6.25, 12.5, 25, 50, 100, and 200 nM) were heated to 95 °C for 5 minutes and cooled slowly to 25 °C. The refolded aptamers were added to the plate with coated proteins and incubated at 25 °C for 30 minutes. Streptavidin-horseradish peroxide was added (1:500) to the wells, followed by incubation at 25 °C for 1 h. TMB (3,3′,5,5′-tetramethylbenzidine) buffer was added to the wells, and the plate was incubated at 25 °C for 20 minutes in the dark. After terminating the reaction with 2 M sulfuric acid, the samples were measured in a plate reader at a wavelength of 450 nm. The Kd value of the nucleic acid aptamer was estimated using the following equation: Y = Bmax ∗ X/(Kd + X). The results were subjected to statistical analysis using GraphPad Prism software.

### 1.6 Gel-shift assay

Aptamers (1 pmol) fluorescently labeled with Fam were heated to 95 °C for 5 minutes, cooled slowly to 25 °C, and incubated with the corresponding ratios of AtCOP1-RING protein in binding buffer for 30 minutes at 25 °C. After adding loading buffer, the samples were separated in 8% non-denaturing PAGE gels. The gels were imaged with a Bio-Rad imaging system with 488 nm excitation.

### 1.7 Imaging

Fluorescence images were acquired under a Leica laser-scanning confocal microscope. Four-day-old *35S-YFP-NLS-COP1/cop1-4* and *35S-GFP/Col-0* seedlings grown in the dark were incubated with 10 μM Cy5-labeled aptamers in DNA Imaging Buffer (20 mM HEPES-KOH pH 7.5, 0.3 mM MgCl_2_, 100 mM KCl) for 12 h at room temperature. For the Cy5 channel, the excitation wavelength was 646 nm, and the emission wavelength range was 663–738 nm. For the YFP channel, the excitation wavelength was 514 nm, and the emission wavelength range was 500–550 nm.

## CRediT authorship contribution statement

**Lihong Song:** Writing – original draft, Formal analysis, Data curation. **Tiantian Zhou:** Validation, Formal analysis, Data curation. **Miqi Xu:** Formal analysis, Data curation. **Kaiqiang Qian:** Formal analysis, Data curation. **Xing Wang Deng:** Writing – review & editing, Project administration, Funding acquisition, Conceptualization. **Qingqing Wu:** Writing – review & editing, Funding acquisition. **Jun-Jie Ling:** Writing – review & editing, Writing – original draft, Supervision, Resources, Funding acquisition, Data curation, Conceptualization.

## Declaration of competing interest

Anhui Agricultural University has filed a patent that includes the work described in this manuscript.

## Data Availability

Data supporting this selection method are available within the article and its Supplementary Information. Sequences of ssDNA libraries, PCR primers and aptamer candidates are listed in Supplementary Table 1–2. Any additional data related to the results reported in this paper are available from the corresponding author upon request.
