# Supplementary material for: One-step selection of high-affinity COP1 aptamers
Source: aBIOTECH. 2026 Jun 4;7(3):100060. doi: 10.1016/j.abiote.2026.100060 (PMC13351135; doi:10.1016/j.abiote.2026.100060)
Supplement: Multimedia component 3 [file mmc3.docx]

**Supplemental Information**

**One-step selection of high-affinity COP1 aptamers**

Lihong Song^a,b,1^, Tiantian Zhou^a,1^, Miqi Xu^a^, Kaiqiang Qian^a^, Xing Wang Deng^b^, and Jun-Jie Ling^a,b,2^

^a^ School of Life Sciences, National Engineering Laboratory of Crop Stress Resistance Breeding, Anhui Agricultural University, Hefei 230036, China.

^b^ State Key Laboratory of Wheat Improvement, Peking University Institute of Advanced Agricultural Sciences, Shandong Laboratory of Advanced Agricultural Sciences at Weifang, Weifang261000, China

^1^ These authors contributed equally to this work

^2^ Correspondence: Jun-Jie Ling, Email: lingjunjie@ahau.edu.cn

**Inventory of Supplemental Information**

Fig. S1 Coomassie brilliant blue (CBB) staining of recombinant AtCOP1-RING.

Fig. S2 Retention profile of aptamer library with indicated time (n = 3).

Fig. S3 PCR detection of aptamer library with indicated time.

Fig. S4 Cladogram of aptamer candidates.

Fig. S5 Retention ratios of aptamer candidates.

Fig. S6 ELONA analysis of Lib2-11 and AtCOP1-RING.

Fig. S7 The analysis of full length of COP1 binding affinity with Lib1-9.

Fig. S8 CBB staining of recombinant COP1-RING proteins from these four species.

Fig. S9 Thrombin-aptamer analysis with ELONA.

Table S1 Top50 of the aptamer candidates.

Table S2 Primers and sequence used in this study.

**
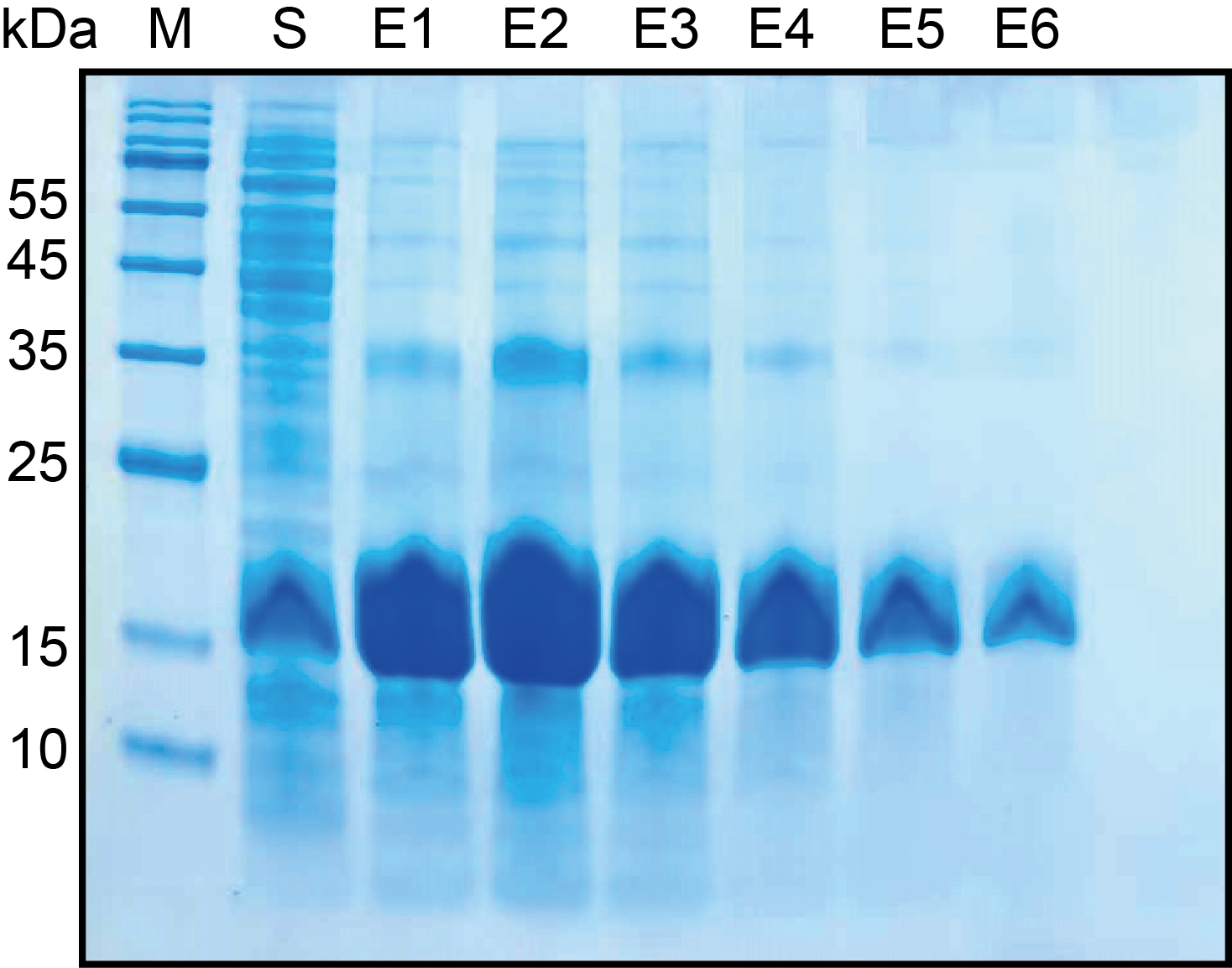
**

**Fig. S1** Coomassie brilliant blue (CBB) staining of recombinant AtCOP1-RING. M, protein marker; S, supernatant; E, elution protein.


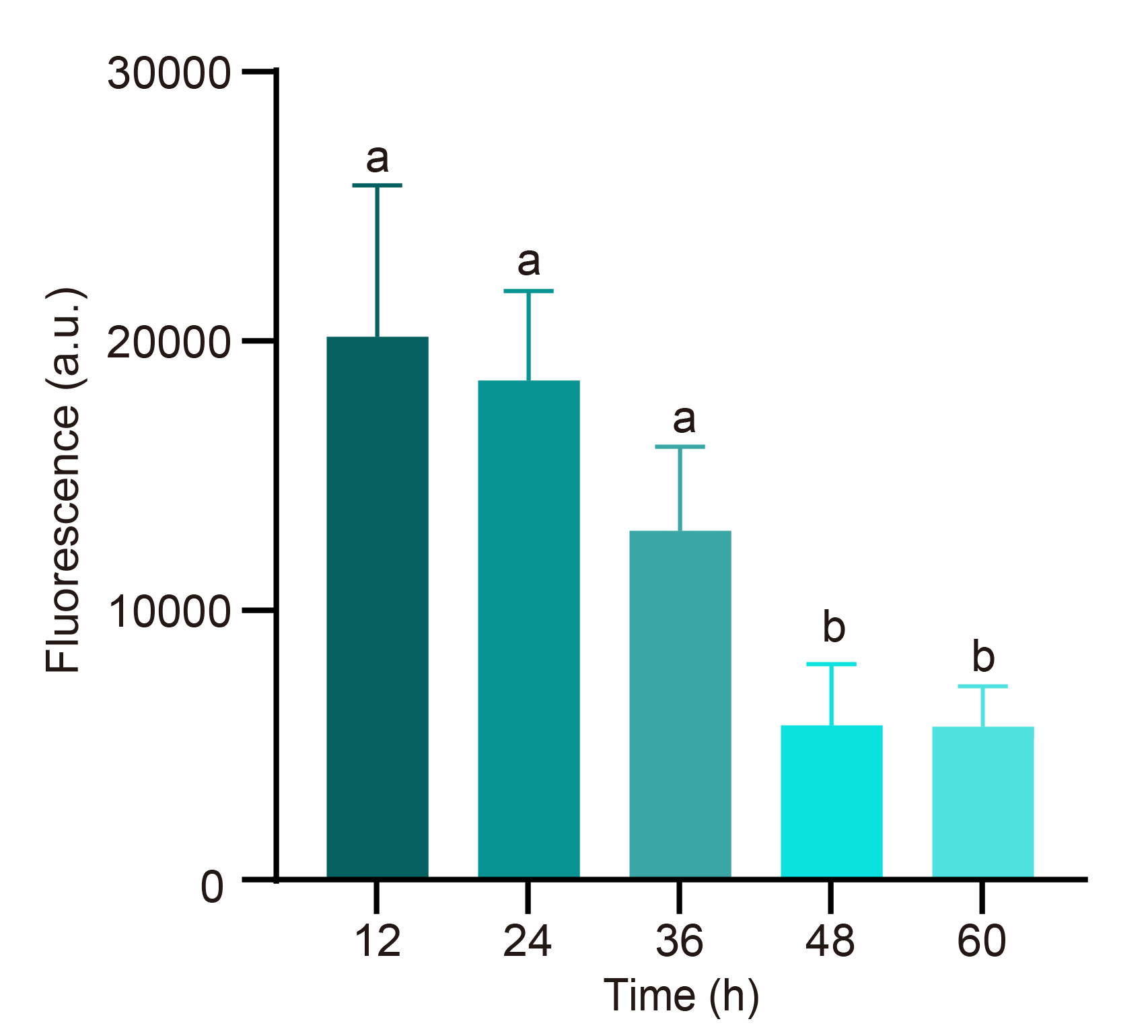


**Fig. S2** Retention profile of Fam-labelled aptamer library with indicated time (n = 3). Data are presented as mean values ± standard deviation (s.d.). The fluorescence of Fam-labelled aptamer library was measured using an Infinite 200 PRO plate reader (TECAN). Statistical analysis was performed by one-way ANOVA with Brown-Forsythe and Welch’s test (significance was set at *P* < 0.05). Different lowercase letters above columns indicate statistical differences at *P* < 0.05.


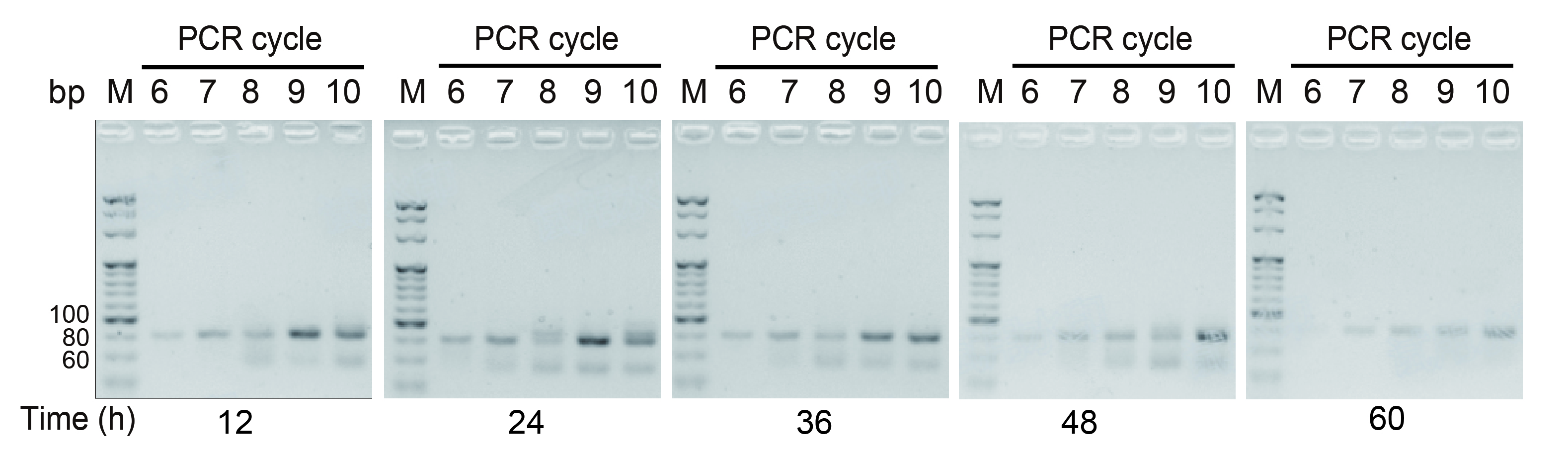


**Fig. S3** PCR detection of aptamer library with indicated time. After elution at the indicated time, the aptamer library was amplified and imaged by UV irradiation. M, DNA marker.


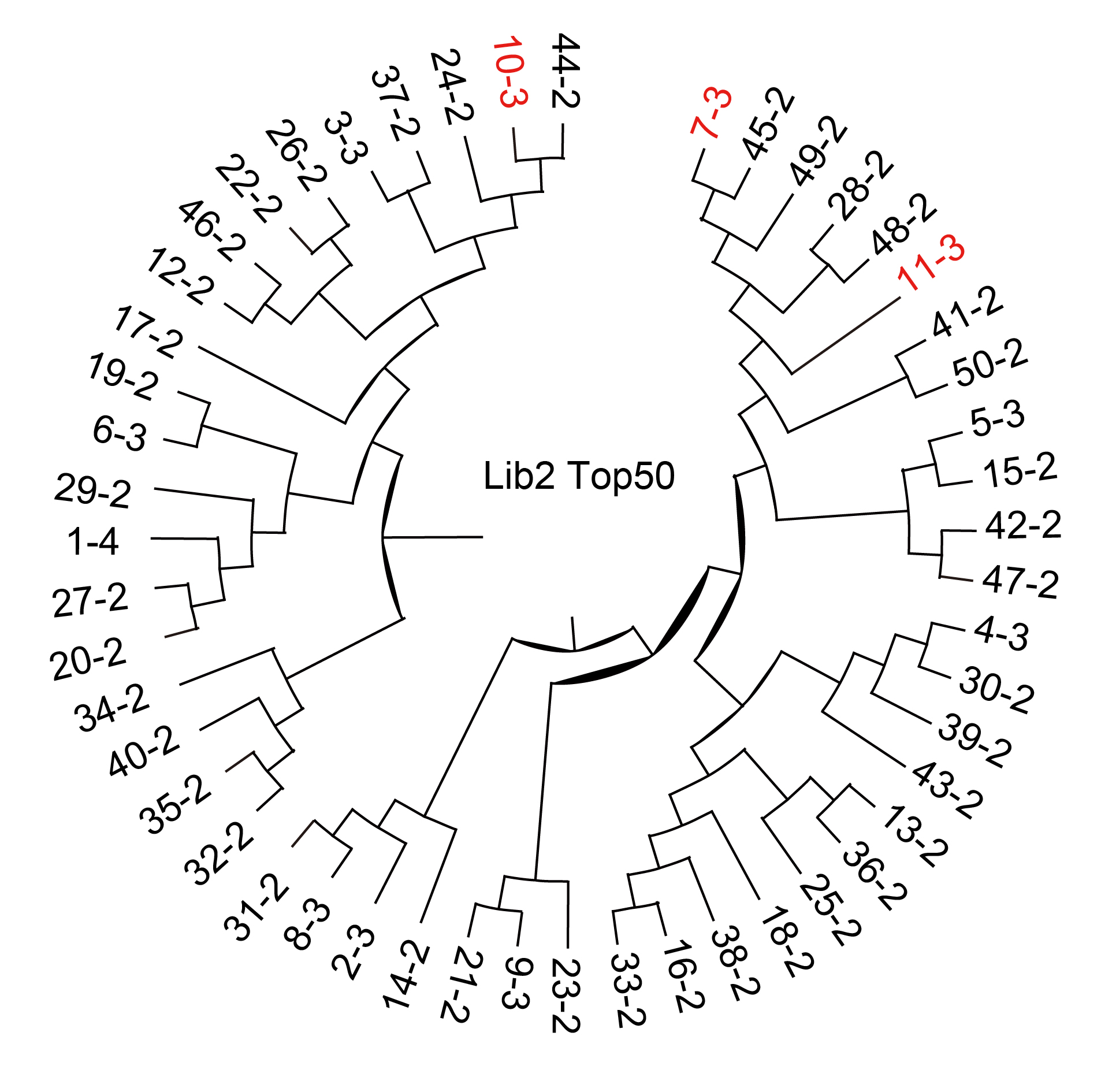


**Fig. S4** Phylogenetic tree of top50 aptamer candidates in Lib2. The candidates with the red color were chosen for further analysis.


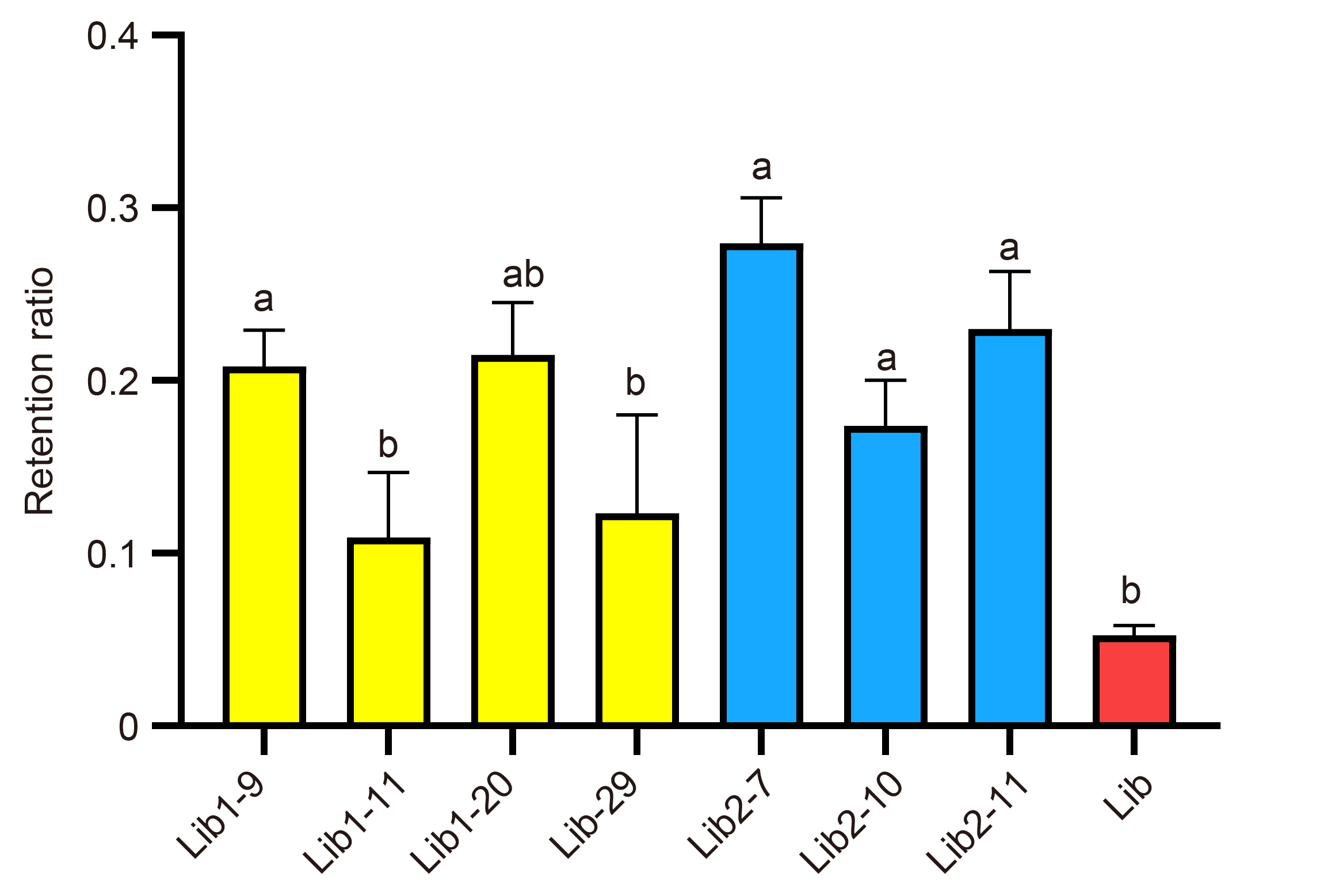


**Fig. S5** Retention ratios of aptamer candidates. Statistical analysis was performed by one-way ANOVA with Brown-Forsythe and Welch’s test (significance was set at *P* < 0.05). Different lowercase letters above columns indicate statistical differences at *P* < 0.05.


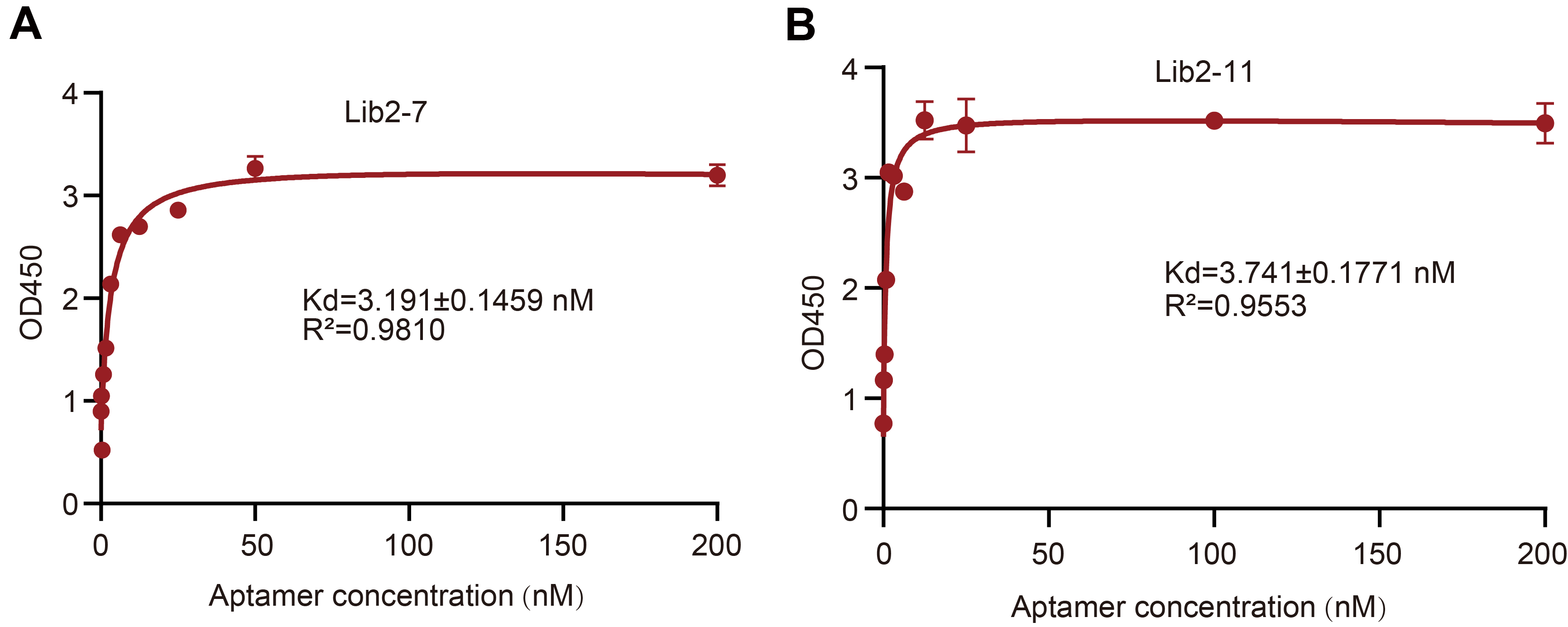


**Fig. S6** ELONA analysis of Lib2-11 and AtCOP1-RING. The aptamer concentrations are 0, 0.20, 0.39, 0.78, 1.56, 3.13, 6.25, 12.5, 25, 50, 100, and 200 nM.


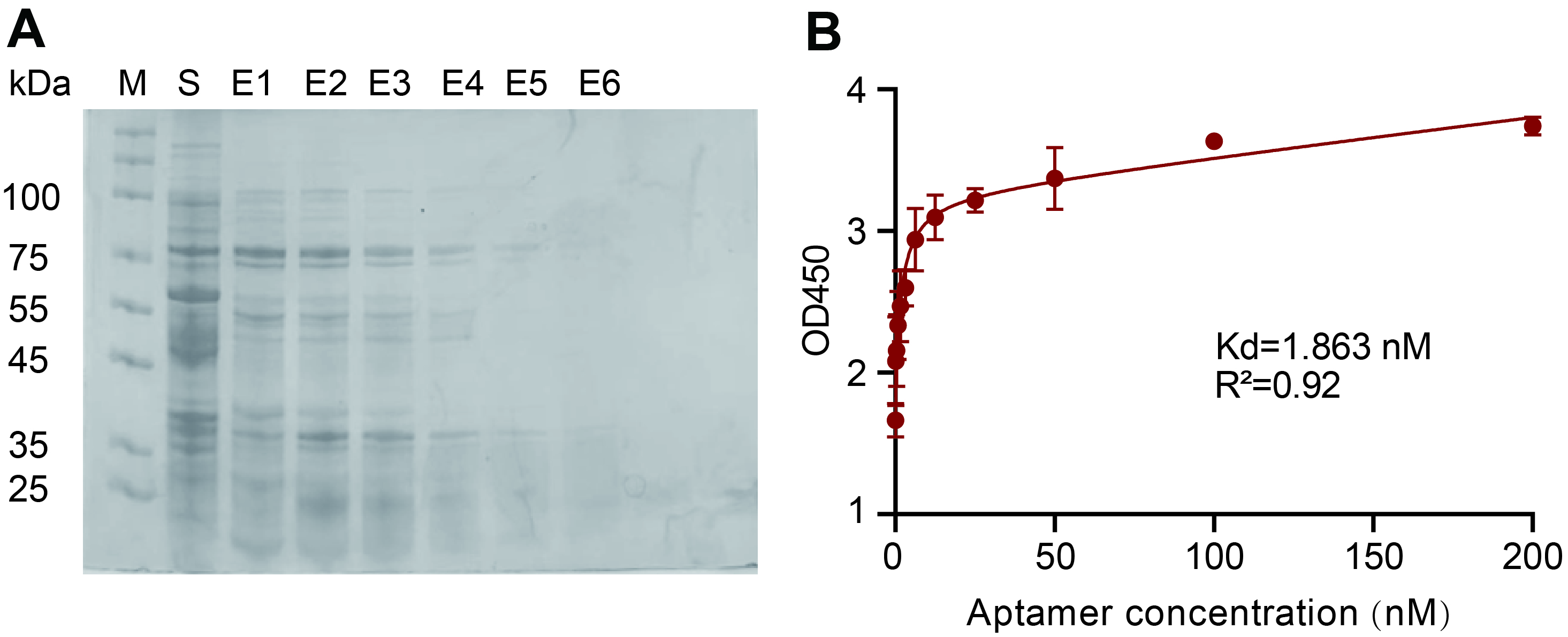


Fig. S7 Binding affinity analysis of Lib1-9 to full-length AtCOP1. **A** CBB staining of recombinant AtCOP1-full length proteins. **B** Binding activity of Lib1-9 and AtCOP1-full length analysis with ELONA. The aptamer concentrations are 0, 0.20, 0.39, 0.78, 1.56, 3.13, 6.25, 12.5, 25, 50, 100, and 200 nM.


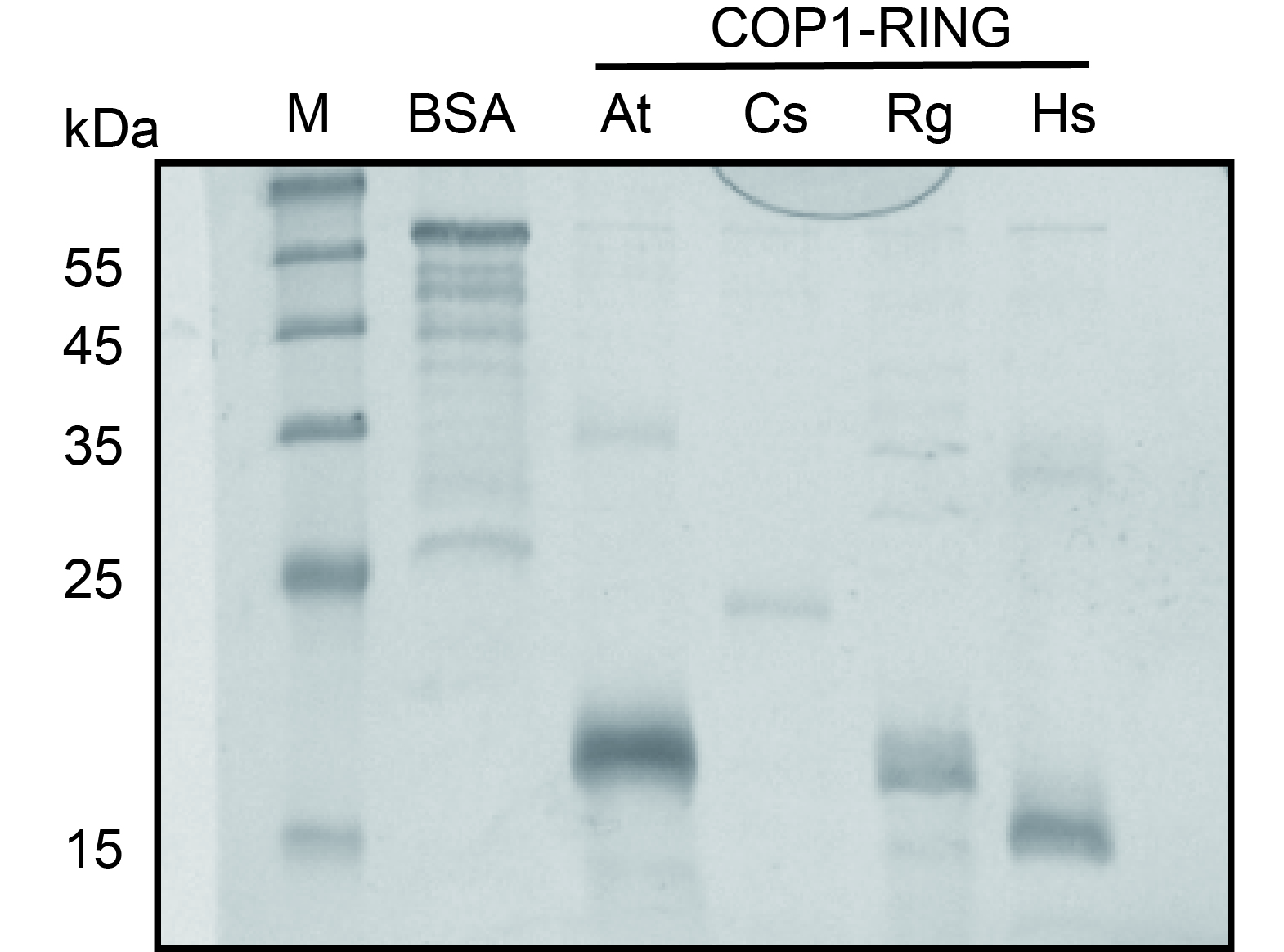


**Fig. S8** CBB staining of recombinant COP1-RING proteins from these four species including *A. thaliana* (At), *R. globosum* (Rg), *C. sinensis* (Cs), and *H. sapiens* (Hs).


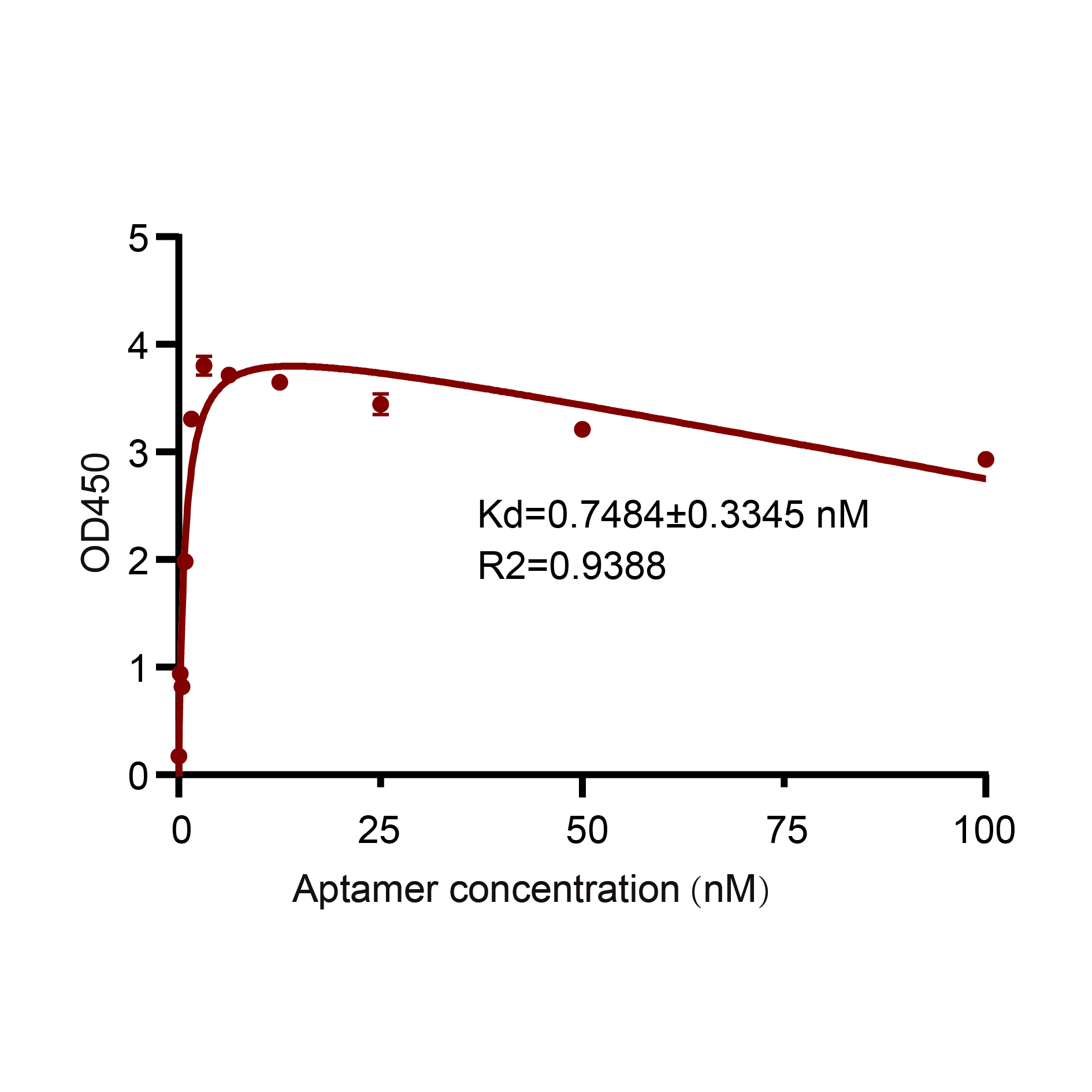


**Fig. S9** Thrombin-aptamer affinity analysis 60.29 with ELONA. The aptamer concentrations are 0, 0.20, 0.39, 0.78, 1.56, 3.13, 6.25, 12.5, 25, 50, and 100 nM.


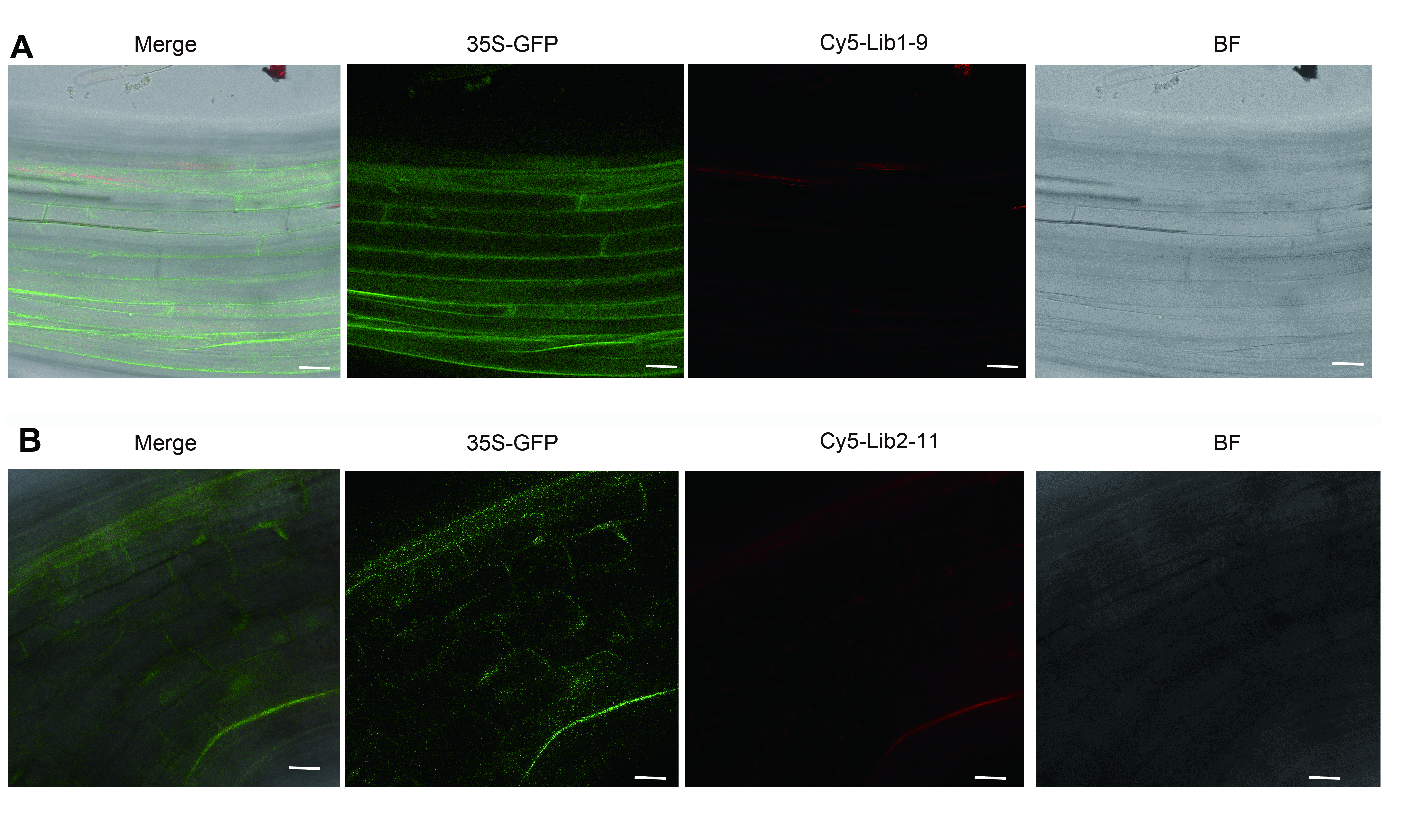


**Fig. S10** Epiﬂuorescence microscopy visualization of Cy5-Lib1-9 (**A**), Cy5-Lib2-11 (**B**) with 35S-GFP. Seedlings were grown in the dark for 4 d and incubated with 10 μM Cy5-Lib2-11 or Cy5-Lib1-9 for 12 h. The scale bar is 10 μm. BF: bright field.
